# The impact of comorbidity on survival after hemorrhagic stroke among dialysis patients: a nationwide population-based study

**DOI:** 10.1186/1471-2369-15-186

**Published:** 2014-11-27

**Authors:** Chun-Yu Lin, Chih-Chiang Chien, Hung-An Chen, Fu-Mei Su, Jhi-Joung Wang, Che-Chuan Wang, Chin-Chen Chu, Yeong-Jang Lin

**Affiliations:** Division of Allergy-Immunology-Rheumatology, Department of Internal Medicine, Chi Mei Medical Center, Tainan, Taiwan; Division of Allergy-Immunology-Rheumatology, Department of Internal Medicine, Chi Mei Medical Center, Liouying, Taiwan; Division of Nephrology, Department of Internal Medicine, Chi Mei Medical Center, Tainan, Taiwan; Department of Food Nutrition, Chung Hwa University of Medical Technology, Tainan, Taiwan; Chia Nan University of Pharmacy and Science, Tainan, Taiwan; Division of Allergy-Immunology-Rheumatology, Department of Internal Medicine, Kaohsiung Chang Gung Memorial Hospital and Chang Gung University College of Medicine, Kaohsiung, Taiwan; Department of Medical Research, Chi Mei Medical Center, Tainan, Taiwan; Department of Neurosurgery, Chi Mei Medical Center, Tainan, Taiwan; Institute of Photonic system, National Chiao Tung University, Tainan, Taiwan; Department of Child Care, Southern Taiwan University of Science and Technology, Tainan, Taiwan; Department of Anesthesiology, Chi-Mei Medical Center, Tainan, Taiwan

**Keywords:** Intracerebral hemorrhage, Dialysis, Comorbidity, Mortality

## Abstract

**Background:**

This study was aimed at determining the outcome and examining the association between comorbidities and mortality after intracerebral hemorrhage in chronic dialysis patients.

**Methods:**

We used the Taiwan National Health Insurance Research Database and enrolled patients who underwent maintenance dialysis between 2000 and 2007. Annual incidence of intracerebral hemorrhage in patients receiving dialysis from 2000 to 2007 was determined. To identify predictors of hemorrhagic stroke, we used logistic regression model to estimate the relative ratio of factors for intracerebral hemorrhage in the most recent cohort (2007). The cumulative survival rate and comorbid conditions associated with mortality after intracerebral hemorrhage among all dialysis patients between 2000 and 2007 was calculated using the Kaplan-Meier method and Cox regression analysis.

**Results:**

We identified 57,261 patients on maintenance dialysis in the cohort of 2007, and 340 patients had history of intracerebral hemorrhage among them. Hypertension was the most common comorbidity of dialysis patients. The incidence rate of intracerebral hemorrhage among dialysis patients was about 0.6%. Adjusted logistic regression model showed that male gender, middle age (45–64 years), hypertension, and previous history of stroke were the independent predictors for the occurrence of intracerebral hemorrhage among chronic dialysis patients. 1,939 dialysis patients with development of intracerebral hemorrhage in the analysis period from 2000 to 2007 were identified. In-hospital mortality was high (36.15%) following intracerebral hemorrhage. They were followed up after intracerebral hemorrhage for a mean time of 41.56 months. Adjusted Cox regression analyses demonstrated that the factors independently associated with mortality after intracerebral hemorrhage among dialysis patients included diabetes mellitus, malignancy and a history of prior stroke.

**Conclusions:**

Dialysis patients who have history of prior stroke, diabetes and malignancy have worse survival than patients without these comorbidities. Attention must focus on providing optimal medical care after hemorrhagic stroke for these target groups to reduce mortality.

**Electronic supplementary material:**

The online version of this article (doi:10.1186/1471-2369-15-186) contains supplementary material, which is available to authorized users.

## Background

Cerebrovascular disease is a common cause of morbidity and mortality in patients with end-stage renal disease (ESRD). Patients with ESRD have advanced atherosclerosis of the cerebral vasculature [1]. Stroke rates for ESRD patients are markedly higher than for the general population [2]. Intracerebral hemorrhage (ICH) is a type of stroke that involves bleeding within the brain tissue itself. It is well known that a bleeding tendency is a major concern among ESRD patients. In addition, the routine administration of heparin during hemodialysis may increase the risk of bleeding. ICH is more common in dialysis patients than in the general population [2], and is a devastating and life-threatening condition that requires urgent intervention. The onset of ICH is usually sudden and, unlike ischemic stroke symptoms, ICH symptoms tend to appear without warning. ICH in dialysis patients has a poor prognosis and a very high mortality rate, despite advances in diagnosis and treatment [3, 4]. It is important to identify dialysis patients at risk for developing ICH and the prognostic factors after ICH.

ICH is more common among Taiwanese than Caucasians [5]. The incidence and prevalence rates of ESRD are also high in Taiwan [6]. However, little is known about the annual incidence of ICH in the Taiwanese dialysis population. There are also few nationwide studies analyzing the predictors of ICH and the effect of comorbidities on survival following ICH in patients on chronic dialysis. In the study, we utilized a large data set from the National Health Insurance Research Database (NHIRD) to identify predicting factors for intracerebral hemorrhage in dialysis patients as the primary objective. The secondary objective was to report the clinical outcomes and identify the independent predictors of ICH mortality in these patients.

## Methods

### Data source

Data for this cohort study was obtained from the NHIRD (http://nhird.nhri.org.tw), which was released for research purposes by the Taiwan National Health Research Institute. The NHIRD, which includes nearly all (99%) of the inpatient and outpatient medical benefit claims for the Taiwanese population of 23 million people, is one of the largest and most comprehensive databases in the world and has been used extensively in various studies [7]. The dataset was released with de-identified secondary data for public research purposes. All types of personal identification on files connected with the present study were scrambled using surrogate identification numbers to secure patient privacy. The study was exempt for the need of written informed patient consent by the Institutional Review Board of Chi-Mei medical center (1008–002) and the Bureau of National Health Insurance (NHRI-NHIRD-99182). We used the NHIRD to collect the ambulatory care claims, all inpatient claims, and the updated registry for beneficiaries from 2000 to 2007 for this study. All datasets can be interlinked through each individual’s unique personal identification number.

### Patient selection and definition

We enrolled ESRD patients in the NHIRD who underwent maintenance dialysis from January 1, 2000 to December 31, 2007. Patients with ESRD are eligible for any type of renal replacement therapy free of charge and without co-payments in Taiwan. Patients on maintenance dialysis were defined as having undergone dialysis longer than 90 days. Patients who had undergone renal transplantation before the initiation of dialysis were excluded. We linked study subjects to the inpatient claims data to identify newly diagnosed ICH (ICD-9-CM code 431).

### Ascertaining the demographic data and comorbidities

Data on age, gender, initial modality, and baseline comorbidities were recorded for each dialysis patient. The baseline comorbidities included diabetes mellitus, hypertension, heart failure, coronary artery disease, old stroke, peripheral arterial disease, chronic lung disease, chronic liver disease, and cancer. Selected comorbidities were ascertained based on 1 in-patient claim or 3 out-patient claims at the start of dialysis or during the follow-up. The ICD-9-CM codes used to define clinical conditions are shown in Additional file 1.

### Statistical analyses

We assessed the annual incidence of ICH among dialysis patients from 2000 to 2007. For each annual cohort, we tabulated all characteristics by presence versus absence of previous ICH, with continuous variables using means and standard deviation and categorical variables using counts and percentages. Because our selected subjects were prevalent dialysis patients, which were not known the time of initiation of dialysis, we used the most recent cohort, for the year 2007, to identify the predictors of ICH and used logistic regression models to estimate the relative ratio for each factor. The cumulative survival rate after developing ICH was calculated using the Kaplan-Meier method. Hazard ratios for each factor were calculated using Cox regression analysis to determine significant predictors for mortality among all dialysis patients with occurrence of ICH between 2000 and 2007. If not addressed, p-values less than 0.05 were considered statistically significant. Data analysis was performed using SPSS for Windows, version 17.0 (SPSS Inc., Illinois, USA).

## Results

### Baseline demographic and clinical data

The patients on maintenance dialysis suffering from ICH accounted for less than 1% of the total dialysis cohort. The majority of patients used hemodialysis as their first modality of renal replacement therapy, but the rate of utilization of peritoneal dialysis as primary treatment for ESRD increased in the analysis period. The prevalence of comorbid conditions in the ESRD population without ICH was substantial: hypertension was the most common, followed by diabetes mellitus, chronic liver disease, coronary artery disease, congestive heart failure, prior stroke, chronic lung disease, cancer, and peripheral artery disease, based on the year 2007 cohort. The frequency of comorbidities among dialysis patients with ICH was somewhat different from those without ICH; i.e., prior stroke was the third most common disease. Moreover, the prevalence of diabetes, hypertension and previous history of stroke among dialysis patients suffering from ICH was even greater. The percentage of patients suffering from ICH in the peritoneal dialysis group was less than in the hemodialysis group (Table 1).

**Table 1:** **Characteristics of patients on maintenance dialysis in selected years between 2000 and 2007 by presence versus absence of ICH**

Characteristics	2000		2004		2007	
	No ICH n = 34282	ICH n = 230	No ICH n = 47555	ICH n = 289	No ICH n = 56921	ICH n = 340
No. (%)						
Age (mean ± SD), y	57.3 ± 14.5	58.9 ± 12	59.1 ± 14.1	58.6 ± 12.5	60.7 ± 13.9	60.7 ± 11.9
Female	18303 (53.4)	107 (46.5)	25598 (53.8)	120 (41.5)	30186 (53.0)	155 (45.6)
Dialysis modality						
Hemodialysis	32340 (94.3)	224 (97.4)	44408 (93.4)	279 (96.5)	52569 (92.4)	320 (94.1)
Peritoneal dialysis	1942 (5.7)	6 (2.6)	2147 (6.6)	10 (3.5)	4352 (7.6)	20 (5.9)
ICH in-hospital days		11.2 (±15.9)		10.9 (±11.3)		13.2 (±15.2)
Comorbid conditions						
Hypertension	17192 (50.1)	143 (62.2)	25044 (52.7)	170 (58.8)	33072 (58.1)	238 (70.0)
Diabetes	11356 (33.1)	101 (43.9)	15897 (33.4)	118 (40.8)	21559 (37.9)	166 (48.8)
Chronic liver disease	7528 (21.9)	26 (11.3)	11902 (25.0)	59 (20.4)	14203 (24.9)	91 (26.8)
Coronary artery disease	6252 (18.3)	48 (20.9)	9290 (19.6)	67 (23.2)	12355 (21.7)	88 (25.9)
Heart failure	3795 (11.1)	33 (14.3)	5734 (12.1)	34 (11.8)	7300 (12.8)	46 (13.5)
Prior stroke	3153 (9.3)	44 (19.1)	4633 (9.8)	63 (21.8)	5815 (10.3)	91 (26.8)
Chronic lung disease	3021 (8.8)	19 (8.3)	3878 (8.2)	24 (8.3)	4209 (7.4)	21 (6.2)
Peripheral artery disease	1858 (5.4)	11 (4.8)	1621 (3.4)	9 (3.1)	2803 (4.9)	27 (8.7)
Cancer	1116 (3.2)	2 (0.9)	2752 (5.8)	13 (4.5)	4150 (7.3)	21 (6.2)

*ICH*, Intracerebral hemorrhage.

### Incidence rate and predictors of ICH in dialysis patients

The incidence rate of ICH in the dialysis population was about 0.6%. 57,261 patients on maintenance dialysis were identified in the most recent and largest cohort of 2007. Results of adjusted logistic regression analysis showed that male gender, middle age (45–64 years at baseline), hypertension, and previous history of stroke were the independent predictors for ICH among chronic dialysis patients. ESRD patients on hemodialysis tended to have a higher incidence of ICH than those on peritoneal dialysis; however, there was no statistical significance (Table 2).

**Table 2:** **Logistic regression model to evaluate for predictors of incidence of intracerebral hemorrhage in patients on maintenance dialysis**

Factors	Univariate	Multivariate
	RR (95% CI)	RR (95% CI)
Sex (Male v Female)	1.35 (1.09-1.67)*	1.25 (1.01-1.55)*
Age		
<44 (Referent)	1	1
45-64	1.86 (1.25-2.77)*	1.56 (1.04-2.34)*
≥ 65	1.44 (0.96-2.17)	1.09 (0.71-1.67)
Dialysis modality (PD v HD)	0.76 (0.48-1.19)	0.81(0.51-1.29)
Comorbidities		
Diabetic mellitus	1.57 (1.26-1.94)*	1.15 (0.91-1.46)
Hypertension	1.68 (1.33-2.12)*	1.37 (1.06-1.77)*
Heart failure	1.06 (0.78-1.45)	0.90 (0.65-1.24)
Coronary artery disease	1.26 (0.99-1.61)	1.04 (0.80-1.35)
Prior stroke	3.21 (2.52-4.09)*	2.86 (2.22-3.69)*
Peripheral arterial disease	1.67 (1.12-2.47)*	1.41 (0.94-2.10)
Chronic lung disease	0.82 (0.53-1.28)	0.74 (0.47-1.16)
Chronic liver disease	1.10 (0.86-1.40)	1.03 (0.81-1.31)
Cancer	0.84 (0.54-1.30)	0.90 (0.57-1.40)

*significant difference.

*PD*, Peritoneal dialysis; *HD*, Hemodialysis; *CI*, Confidence interval; *RR*, Relative ratio.

### Predictors for all-cause mortality after ICH in dialysis patients

**Figure 1:**
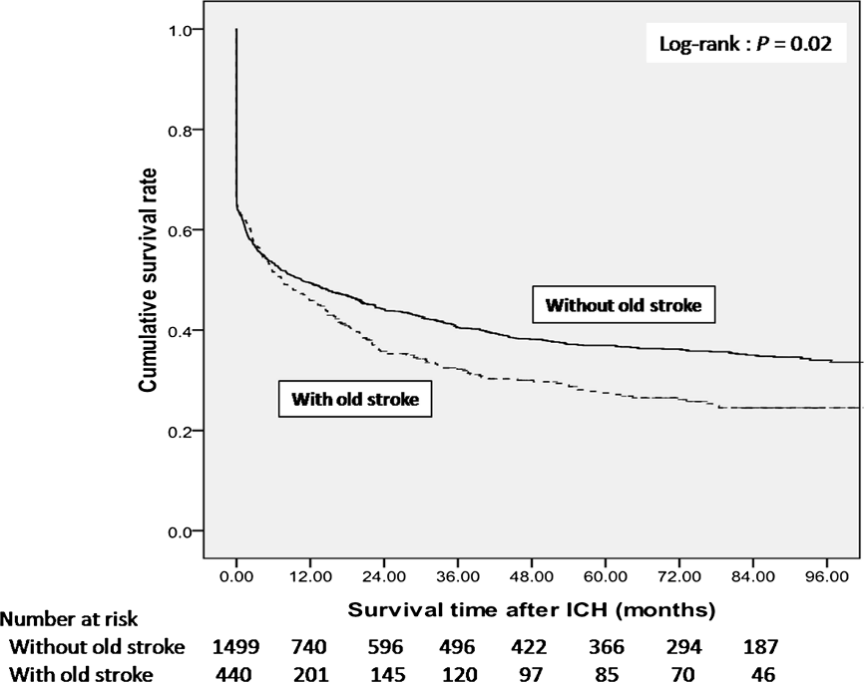
**Crude overall survival curves after intracerebral hemorrhage among dialysis patients stratified as being with and without prior stroke.**

1,939 dialysis patients with the occurrence of intracerebral hemorrhage from 2000 to 2007 were identified. The mean of follow-up time after ICH was 41.56 months. Overall, we found a high in-hospital mortality rate (36.15%) among dialysis patients hospitalized with ICH. In the univariate analysis of baseline data, diabetes mellitus, hypertension, coronary artery disease, previous history of stroke, chronic liver disease, and malignancy were associated with higher mortality following ICH hospitalization. The multivariate Cox regression analysis showed that previous history of stroke, diabetes mellitus and malignancy were independent predictors of mortality after ICH among dialysis patients. The in-hospital mortality rate in patients with a prior history of stroke was almost the same as in patients without a prior history of stoke (36.14% vs. 36.16%). Crude overall survival curves after intracerebral hemorrhage among dialysis patients with and without prior stroke were shown in the Figure 1. The two survival curves started to separate at about 8 weeks after ICH. The crude survival rates at 1 year and 2 years after ICH differed between dialysis patients with and without a history of prior stroke (45.7% vs. 49.4%, 35.5% vs. 44.1% respectively) (log rank: *P* = 0.02) (Figure 1) (Table 3).

**Table 3:** **Cox regression model to evaluate for predictors of all-cause mortality after intracerebral hemorrhage in dialysis patients**

Factors	Univariate analysis	Multivariate analysis
	HR (95% CI)	HR (95% CI)
Sex (male v female)	1.07 (0.96-1.20)	1.08 (0.96-1.20)
Age		
<45	1	1
45-64	1.05 (0.86-1.27)	1.01 (0.82-1.23)
≥ 65	1.20 (0.98-1.47)	1.13 (0.91-1.39)
Dialysis modality (PD v HD)	0.97 (0.72-1.32)	1.02 (0.75-1.39)
Comorbidities		
Diabetic mellitus (yes v no)	1.15 (1.03-1.29)*	1.14 (1.01-1.28)*
Hypertension (yes v no)	1.06 (0.94-1.18)	0.95 (0.83-1.08)
Heart failure (yes v no)	0.97 (0.82-1.15)	0.93 (0.78-1.11)
Coronary artery disease (yes v no)	1.15 (1.01-1.31)*	1.09 (0.95-1.26)
Prior stroke (yes v no)	1.20 (1.05-1.36)*	1.17 (1.03-1.34)*
Peripheral arterial disease (yes v no)	1.11 (0.87-1.43)	1.05 (0.82-1.35)
Chronic lung disease (yes v no)	1.07 (0.87-1.32)	1.01 (0.82-1.26)
Chronic liver disease (yes v no)	1.17 (1.02-1.33)*	1.12 (0.98-1.28)
Cancer (yes v no)	1.79 (1.39-2.31)*	1.74 (1.34-2.25)*

*significant difference.

*PD*, Peritoneal dialysis; *HD*, Hemodialysis; *HR*, Hazard ratio; *CI*, Confidence interval.

## Discussion

This nationwide study was designed to investigate the risk factors of ICH occurrence and predictors of mortality following ICH in prevalent dialysis patients using the Taiwan National Health Insurance (NHI) database. We found a high incidence rate of ICH among chronic dialysis patients. Male gender, middle age, hypertension, and history of old stroke were independent factors of incident ICH. Diabetes mellitus and malignancy were significant independent predictors of mortality after ICH. Long-term outcome of ICH survivors was much poorer among the group with a history of prior stroke than among those without a history of prior stroke.

The present findings confirm the high rate of ICH among dialysis patients compared with the general population reported in a prior study [2]. We also showed that hypertension and male gender were independent predictors of hemorrhagic stroke among patients on chronic dialysis. These observations were consistent with recent study by Yoo et al., who evaluated predictive variables of ICH in dialysis patients using the United States Renal Data System (USRDS) database [8]. Similar results were also observed in previous study by Seliger’s [9], in which increased ICH rate was associated with a raised mean arterial pressure of at least >10 mmHg. Thus, hypertension is a risk factor for ICH for both the general population and dialysis patients [10]. While higher blood pressure has relevance to incidental ICH, the effect of it on all-cause mortality seems to be different. We did not find an association between hypertension and all-cause mortality after ICH in patients undergoing chronic dialysis. Our finding that a history of hypertension is not an independent predictor of death among chronic dialysis patients is in agreement with the work of Tetri and colleagues, who investigated the factors associated with outcome after ICH in the general population [11]. However, the observation of a null association between elevated blood pressure and poor outcome of dialysis patients should be interpreted with some caution. We did not differentiate patients using antihypertensive medications from those without treatment in this analysis, and it seems probable that the effect of hypertension is confounded by the use of these drugs.

This study found that previously diagnosed diabetes predicted mortality. Diabetes was also demonstrated to be an independent determinant of death in the general population suffering from ICH [12]. The exact reason why diabetes is associated with the risk of mortality following ICH remains unclear. The effect of DM on survival may be explained by the severity of hemorrhagic events. Diabetes induces structural and functional changes in the microvasculature that lead to fibrinoid necrosis of blood vessel walls and microaneurysm formation. It is possible that these changes in vessel walls make diabetic patients more likely to develop larger sized hematomas than non-diabetics. There is evidence showing that patients with an elevated glucose level due to diabetes were associated with hemorrhages of a larger size compared to patients with a normal glucose level [13], and that the volume of ICH was a strong predictor of mortality after primary ICH [14, 15]. In addition, patients with uncontrolled diabetes and a high glucose level may also have a higher risk of infectious or cardiovascular complications during hospitalization.

Moreover, we found that a previous medical history of stroke is strongly associated with both a higher incidence of ICH and higher mortality risk following ICH among dialysis patients. The risk of ICH was reported to increase several times after ischemic infarct in the general population [16, 17]. It is well established that dialysis patients have a much higher incidence of cerebral ischemic events. Therefore, it is prudent to avoid over-anticoagulation in these patients and to monitor them more closely when providing antithrombotic drugs to prevent recurrent ischemic infarction. A complex series of pathophysiological events are initiated in the brain after ischemic injury. It has been reported that matrix metalloproteinases-2 (MMP-2), MMP-3 and MMP-9, which are capable of digesting basal lamina and extracellular matrix components and lead to subsequent blood brain barrier breakdown and hemorrhagic transformation, were abnormally upregulated in the ischemic region and peri-infarct area [18–20]. Increased expression of MMP-9 of the brain tissue was also observed in patients suffering from hemorrhagic stroke [20]. Moreover, the increased activities of inducible matrix proteases may sustain for several months or years after brain injury [21]. It is possible that physiologic and pathologic responses to stroke cause chronic damage to vessel wall components and pose a sustained risk of subsequent ICH among dialysis patients.

The present study showed the in-hospital mortality rate among dialysis patients hospitalized with ICH was 36.15%. This result correlated with previous reports from other Asian countries. In a study from Korea, the 30-day mortality rate among 102 ESRD cases of spontaneous ICH was 53.9% [22]. Another study from Japan using registered data showed that the death rate of dialysis patients after cerebral hemorrhage was 27.9% [23]. A recent study from Taiwan reported that the mortality rate within 30 days after spontaneous ICH in the general population was 19.8% [24]. Thus, the outcome of ICH is poor among patients on chronic dialysis, and can be worse than in the general population.

Whether gender has an impact on survival after stroke remains controversial. It has been suggested that estrogen may exert neuroprotective effects after brain injury by way of stimulating insulin-like growth factor-I synthesis, lessening inflammatory processes, scavenging free radicals, and stimulation of intrinsic anti-apoptotic pathways [25, 26]. A recent study demonstrated a significant survival advantage for females over males over 3-year period after primary ICH [27]. Another study in Denmark also revealed a female superiority in survival time after stroke [28]. However, we did not find such an association between female gender and lower all-cause mortality rates after hemorrhagic stroke among chronic dialysis patients.

Peritoneal dialysis (PD) was claimed to have some benefits, such as minimal anticoagulation and improved cardiovascular or intracranial stability as a consequence of smaller changes in serum osmolality. There is also evidence showing that intracranial pressure increased significantly in patients receiving conventional intermittent hemodialysis [29]. So patients on PD were expected to experience fewer acute cerebral events compared with those on hemodialysis (HD), and some authors suggest a survival advantage with the use of PD instead of HD for chronic dialysis patients with cerebral hemorrhage [30, 31]. In the USRDS study by Yoo mentioned above, use of peritoneal dialysis as long-term renal replacement therapy was associated with lower risk of ICH compared to use of hemodialysis [8]. However, there is a contradictory result. A recent work showed an even worse prognosis for patients receiving PD after ICH [4]. Our analysis did not support the association of PD as a dialysis modality with less incidental ICH or decreased mortality following ICH among chronic dialysis patients.

The strength of our study lies in its large population size, and high likelihood that nearly all relevant patients were collected from the database. The limitations of this study include a lack of laboratory data, and a lack of information about the causes of death, because they were not included in the Taiwan NHI database. We used ICD-9 code 431 to identify cases of ICH, so patients with isolated subarachnoid hemorrhage (ICD-9 code: 430) would not be included in our analysis. We could not affirmatively determine the duration and severity of comorbidities, which may influence outcome of our subjects, in the databases-derived study. We did not have access to detailed information about ICH and dialysis, so the severity and location of the ICH, blood pressure values, and dialysis adequacy cannot be assessed. Surgery was required in patients with large hematoma formation or refractory elevated intracranial pressure. Operation may have an influence on short-term mortality or long-term disability in our study subjects but we did not analyze the proportion of patients receiving surgical treatment after ICH. Moreover, we did not evaluate drug treatment in ESRD population, such as antiplatelet or anticoagulants, which may be associated with incidence of ICH. We also did not differentiate ischemic stroke from hemorrhagic stroke in determining comorbidities. Further large prospective studies with detailed evaluations are needed to confirm our findings.

## Conclusion

In summary, our nationwide study showed that patients on chronic dialysis had a high risk of hemorrhagic stroke. The factors associated with ICH occurrence and post-hemorrhage survival of these patients were somewhat different from the traditionally reported risk factors for ICH. Dialysis patients that have history of prior stroke, diabetes or malignancy have worse survival than patients without these comorbidities. It is important to identify patients with modifiable risks; this will enable clinicians to provide the patients with preventive strategies and best medical care to achieve better outcomes during the dialysis period.

## Electronic supplementary material

Additional file 1:**ICD-9-CM codes used to identify clinical conditions.**(DOC 29 KB)
