# Supplementary material for: The impact of comorbidity on survival after hemorrhagic stroke among dialysis patients: a nationwide population-based study
Source: BMC Nephrol. 2014 Nov 27;15:186. doi: 10.1186/1471-2369-15-186 (PMC4256891; doi:10.1186/1471-2369-15-186)
Supplement: Supplementary file 1 — Additional file 1:ICD-9-CM codes used to identify clinical conditions.(DOC 29 KB) [file 12882_2014_873_MOESM1_ESM.doc]

***Additional file 1*. ICD-9-CM codes used to identify clinical conditions**

| **Conditions** | ***ICD-9-CM*** |
| --- | --- |
| Intracerebral hemorrhage | 431 |
| Diabetes | 250.**, 357.2, 362.0*, 366.41 |
| Hypertension | 362.11, 401.*-405.*, 437.2 |
| Heart failure | 428.0-428.43, 428.9, 398.91 |
| Coronary artery disease | 410.**- 414.** |
| Stroke | 430-438.** |
| Peripheral arterial disease | 440.0-440.9, 38.13-38.18, 39.22-39.26, 39.28 |
| Chronic lung disease | 490-496*, 500-505*, 506.4* |
| Chronic liver disease | 571.2*, 571.5*, 571.6*, 571.4-571.49* |
| Cancer | 140.**-208.** |

ICD-9-CM, *International Classification of Diseases, Ninth Revision, Clinical Modification*; TIA, transient ischemic attack.

* can be any number or missing.
